# Transcriptome and Expression Profile Analysis of Highly Resistant and Susceptible Banana Roots Challenged with *Fusarium oxysporum* f. sp. *cubense* Tropical Race 4

**DOI:** 10.1371/journal.pone.0073945

**Published:** 2013-09-23

**Authors:** Ting-Ting Bai, Wan-Bin Xie, Ping-Ping Zhou, Zi-Lin Wu, Wen-Chao Xiao, Ling Zhou, Jie Sun, Xiao-Lei Ruan, Hua-Ping Li

**Affiliations:** 1 State Key Laboratory of Conservation and Utilization of Subtropical Agro-bioresources, South China Agricultural University, Guangzhou, Guangdong, China; 2 College of Natural Resources and Environment, South China Agricultural University, Guangzhou, Guangdong, China; Nanjing Agricultural University, China

## Abstract

Banana wilt disease, caused by the fungal pathogen *Fusarium oxysporum* f. sp. *cubense* 4 (Foc4), is regarded as one of the most devastating diseases worldwide. Cavendish cultivar ‘Yueyoukang 1’ was shown to have significantly lower disease severity and incidence compared with susceptible cultivar ‘Brazilian’ in greenhouse and field trials. *De novo* sequencing technology was previously performed to investigate defense mechanism in middle resistant ‘Nongke No 1’ banana, but not in highly resistant cultivar ‘Yueyoukang 1’. To gain more insights into the resistance mechanism in banana against Foc4, Illumina Solexa sequencing technology was utilized to perform transcriptome sequencing of ‘Yueyoukang 1’ and ‘Brazilian’ and characterize gene expression profile changes in the both two cultivars at days 0.5, 1, 3, 5 and 10 after infection with Foc4. The results showed that more massive transcriptional reprogramming occurs due to Foc4 treatment in ‘Yueyoukang 1’ than ‘Brazilian’, especially at the first three time points, which suggested that ‘Yueyoukang 1’ had much faster defense response against Foc4 infection than ‘Brazilian’. Expression patterns of genes involved in ‘Plant-pathogen interaction’ and ‘Plant hormone signal transduction’ pathways were analyzed and compared between the two cultivars. Defense genes associated with CEBiP, BAK1, NB-LRR proteins, PR proteins, transcription factor and cell wall lignification were expressed stronger in ‘Yueyoukang 1’ than ‘Brazilian’, indicating that these genes play important roles in banana against Foc4 infection. However, genes related to hypersensitive reaction (HR) and senescence were up-regulated in ‘Brazilian’ but down-regulated in ‘Yueyoukang 1’, which suggested that HR and senescence may contribute to Foc4 infection. In addition, the resistance mechanism in highly resistant ‘Yueyoukang 1’ was found to differ from that in middle resistant ‘Nongke No 1’ banana. These results explain the resistance in the highly resistant cultivar and provide more insights in understanding the compatible and incompatible interactions between banana and Foc4.

## Introduction

*Musa spp.* (bananas and plantains) is one of the most important fruit crops in the world and their global annual production account to more than 120 Mt [1]. *Musa* crop not only has the prominence as a dessert fruit, but also provides a vital source of food to many inhabitants of the humid tropics. Banana cultivation, like that of all other crop species, is affected by distinct constraints, among which Fusarium wilt caused by *Fusarium oxysporum* f. sp. *cubense* (Foc) is considered to be one of the most important threats [2]. Fusarium wilt of banana also known as Panama disease was first reported from Panama as early as 1890. The disease had seriously affected banana cultivation for more than 60 years in tropical America of the last century. And it was under control only when the susceptible cultivar Gros Michel was replaced by the resistant Cavendish banana cultivars [3]. Since then, Cavendish type of banana becomes the major cultivars and the international export trade has converted from the susceptible cv. Gros Michel to the resistant cv. Cavendish [4]. However, Foc4, a new race of Foc, is found to be able to infect Cavendish cultivars and has caused great damage to Cavendish production worldwide in recent years [2].

As *Fusarium oxysporum*, Foc4 is a typical necrotrophic fungus [5], and it can survive in soil for long periods in the form of thick-walled chlamydospores before infecting banana [6]. Foc4 penetrates banana roots through small openings or wounds in the cell wall [7], and kills host cells and tissue to derive nutrition from the dead cells. Then the fungus proliferates within the vascular tissue, disrupts water translocation and causes the typical wilt symptoms, which include foliage cholrosis, pseudostem longitudinal splitting, necrosis and ultimate death [8]. Foc4 is divided into two types: subtropical race 4 (ST4) affecting Cavendish production in subtropical areas like Taiwan, Canary Islands, South Africa and Australia and tropical race 4 (TR4) found in the tropical regions of Southeast Asia and Australia. The control measures of Fusarium wilt disease mainly include destruction of diseased plants, use of disease-free plant materials, soil rotation with paddy rice and use of fungicide. However, all of these control methods still cannot prevent banana from Foc4 infection effectively. As a result of triploid varieties with highly sterility, it is very hard to develop wilt-resistant banana cultivars by traditional breeding method. At the present time, the best way to control Fusarium wilt disease is based on molecular breeding for resistant cultivars.

Currently genomics and disease-resistance genomics resources of banana that are available online are very limited. The lack of massive genomics resources is one of the major constraints in banana wilt-resistant breeding. Recently the rapid, low-costing next generation sequencing (high-throughput deep sequencing) technology has become a powerful tool for discovery of novel gene, expression profiling, comparative genomics study and evolutionary genomics research [9]. In the past few years, *de novo* sequencing technology has been successfully used for molecular mechanism investigation of plants after pathogen infection, such as grape [10], tobacco [11], wheat [12], and so on. For banana, Wang characterized root transcriptome of the Foc4-susceptible cultivar ‘Brazilian’ and investigated the transcriptional changes in banana roots 2, 4 and 6 days post infection (DPI) [13]. Li compared the gene expression profiles of the middle resistant cultivar ‘Nongke No 1’ and the susceptible cultivar ‘Brazilian’ infected with Foc4 at 2 and 4 days [14], [15]. However, little is known about transcriptional changes in roots of Foc4-challenged banana 0–2 DPI or after 6 DPI. And it is noteworthy that related research in highly wilt-resistant cultivar still remains undone.

To obtain more transcriptome information of banana and further understand the molecular mechanism of the banana resistance against wilt disease in highly resistant cultivar, we performed transcriptome sequencing of the highly resistant ‘Yueyoukang 1’ banana using Illumina technology. A total of 87,845 unigenes were obtained and subsequently served as reference database for gene expression profile analysis. KEGG annotation revealed that ‘Plant-pathogen interaction’ and ‘plant hormone signal transduction’ pathway respectively included 2579 and 2696 genes. We further monitored gene expression profile changes in both ‘Yueyoukang 1’ and ‘Brazilian’ at time points 0.5, 1, 3, 5 and 10 days post Foc4 infection. The results showed that the number of differentially expressed genes in highly resistant ‘Yueyoukang 1’ was far more than that in susceptible ‘Brazilian’ at the first three infection time points. It was interesting that the expression patterns of many defense genes involved in PAMP-triggered immunity (PTI), effector-triggered immunity (ETI), regulation of ion influx and cell wall reinforcement were different between ‘Yueyoukang 1’ and ‘Brazilian’ challenged with Foc4. The study first investigates the resistance mechanism in highly resistant banana against Foc4 using the Illumina Solexa sequencing technology and provides more insights into the understanding of banana-Foc interaction.

## Materials and Methods

### Plant material and pathogen inoculation

‘Brazilian’ (AAA, Cavendish) is a cultivar susceptible to Foc4, and ‘Yueyoukang 1’ (AAA, Cavendish) is a cultivar highly resistant to Foc4 [16]. Before the research, we performed inoculation experiments with Foc TR4 and confirmed that ‘Brazilian’ and ‘Yueyoukang 1’ were susceptible and highly resistant cultivar, respectively. Tissue cultured banana plants (cv. ‘Brazilian’ and cv. ‘Yueyoukang 1’) were transplanted into 100-mm-diameter pots with sterile peat-coconut coir mix. They were distributed at random in an incubator at 28°C with a 16-h light/8-h dark photoperiod. After 8 weeks, plants with five leaves and healthy root system were chosen for TR4 inoculation. Their roots were soaked in a TR4 spore suspension of 10^6^ condia/ml for 30 minutes and then were planted back the pots. The entire root system was harvested at time points 0.5, 1, 3, 5 and 10 days post infection (DPI), flash frozen in liquid nitrogen and stored at −70°C. Nine plants were used for each time point and all collection roots of the nine plants were pooled together. The roots harvested from the uninfected banana plants at 0 day as described above were served as a control.

### RNA extraction and library preparation for transcriptome sequencing

Total RNA was extracted from roots of cv. ‘Brazilian’ and cv. ‘Yueyoukang 1’ using the QIAGEN RNeasy plant mini kit (QIAGEN, Valencia, CA), respectively, and treated with RNase-free DNAse I (Promega, Madison, Wisconsin, USA). RNA integrity was confirmed using the 2100 Bioanalyzer (Agilent Technologies) with a minimum RNA integrated number value of 8. The mixture of equal amounts of the two cultivar root RNA samples for transcriptome analysis were prepared using Illumina's kit following manufacturer's recommendations. Briefly, mRNA was purified from 20 µg of total RNA using Sera-mag Magnetic Oligo (dT) Beads (Illumina) and then was fragmented into small pieces using divalent cations under elevated temperature. The cleaved RNA fragments were used for double-stranded cDNA synthesis using double-stranded cDNA Synthesis kit (Invitrogen, Camarillo, CA) with random hexamer (N6) primers (Illumina). These cDNA fragments then went through an end repair process, phosphorylation, and ligation of adapters. Then these products were purified and enriched with PCR to create the final cDNA library.

### Transcriptome sequencing, assembly and annotation

The cDNA library prepared was sequenced on the Illumina HiSeq™ 2000. And both ends of the cDNA were sequenced. The 90 bp pair-end raw reads were generated by the Illumina Genome Analyzer II system. Image deconvolution and quality value calculations were performed using the Illumina GA pipeline 1.3. Clean reads were obtained by moving the empty reads, the adaptor sequences, and the low-quality sequences (reads with unknown base pairs ‘N’). The clean reads were then assembled into contigs and scaffolds based on pair-end information using SOAPdenovo. [17]. Finally, gaps of the scaffolds were filled using paired-end to obtain unigenes which contained the least number of Ns and could not be extended on either end.

Annotation of the unigenes was performed by running our assembly against protein databases including NCBI nr with a cut-off E-value of 10^−5^, KEGG and COG. The proteins from NCBI nr database with the highest sequence similarity to the unigenes were used to assign functional annotations to the unigenes [18]. Gene ontology (GO) annotation was carried out using the BLAST2GO program based on unigenes annotation from nr [19], [20]. The KEGG pathway and COG annotation was performed using BLASTALL software [21], [22].

### Digital gene expression (DGE) library preparation and sequencing

Total RNA was respectively extracted from roots of cv. ‘Brazilian’ and cv. ‘Yueyoukang 1’ after TR4 infection 0, 0.5, 1, 3, 5, and 10 DPI using the QIAGEN RNeasy plant mini kit (QIAGEN, Valencia, CA). DGE libraries were prepared using the Illumina gene expression sample prep kit. Briefly, mRNA was purified from 6 µg of total RNA using oligo(dT) magnetic beads. Double-stranded cDNA were directly synthesized on the RNA-bound beads and was subsequently digested with NlaIII. The fragmentized cDNAs which contained 3′ ends were washed away the magnetic beads, and then the Illumina adaptor 1 with an additional restriction site of Mme I was ligated to the 5′ ends of these cDNA fragments. After digestion with Mme I and CATG site, 21–22 bp tags containing the adaptor 1 sequence were released. Then the Illumina adaptor 2 was added to the 3′ end of the tags to produce a tag library. After 15 cycles of PCR amplification, 85 bp strips were purified from 6% PAGE gels. The cDNAs were digested, and the single-stranded molecules were attached to the Illumina sequencing chip for sequencing.

### Analysis and mapping of DGE tags

Low quality tags (tags with unknown nucleotide “N”), empty tags (no tag sequence between the adaptors) and tags with only one copy number (which might result from sequencing errors) were removed from the DGE tags to generate clean tags. In order to annotate the tags, the clean tags containing CATG and 21 bp tag sequences were mapped to our transcriptome reference database, allowing no more than one nucleotide mismatch. All the tags mapped to reference sequences from multiple genes were filtered and the remaining tags were designated as unambiguous tags. For gene expression analysis, the number of expressed tags was calculated and normalized to the number of transcripts per million (TPM) tags. The differentially expressed tags were used for further mapping and annotation.

### Quantitative real-time PCR (qRT-PCR) validation

Total RNA was extracted as described for the transcriptome library preparation and sequencing. RNA was reverse transcribed in a 20 µl reaction system using the PrimeScript™ RT Master Mix Kit (TaKaRa). Gene-specific primers were designed base on the gene sequences using Primer 5.0 software and the primer sequences are listed in Table S1. The rps2 gene of banana was used as a reference gene [23]. The qRT-PCR was performed using the SYBR Premix Ex Taq Kit (TaKaRa) according to the manufacturer's protocol. And all qRT-PCR reactions were carried out using Thermal Cycler Dice (Takara, Japan). Individual reactions were run with each primer pair with annealing temperatures ranging from 55°C to 60°C. The conditions were as follows: initial holding at 95°C for 30 s, followed by a two-step program of 95°C for 15 s and annealing temperature for 30 s for 40 cycles. Each sample was analyzed in three technical replicates. The relative changes in gene expression levels were calculated using the 2^−ΔΔCt^ method.

### Statistical analysis

The differentially expressed genes (DEGs) of cv. ‘Brazilian’ and cv. ‘Yueyoukang 1’ in different TR4 infection stages (0, 0.5, 1, 3, 5, and 10 DPI) were identified using an algorithm developed according to “The significance of digital gene expression profiles” described by Audic et al [24]. The false discovery rate (FDR) was used to determine the threshold of P value in multiple test and analysis. A threshold of FDR<0.001 was used to judge the significance of gene expression difference. We used “FDR≤0.001 and the absolute value of fold change (log2Ratio)≥1” as the threshold to judge the significance of gene expression difference [25].

## Results

### Illumina sequencing and sequence assembly

**Figure 1:**
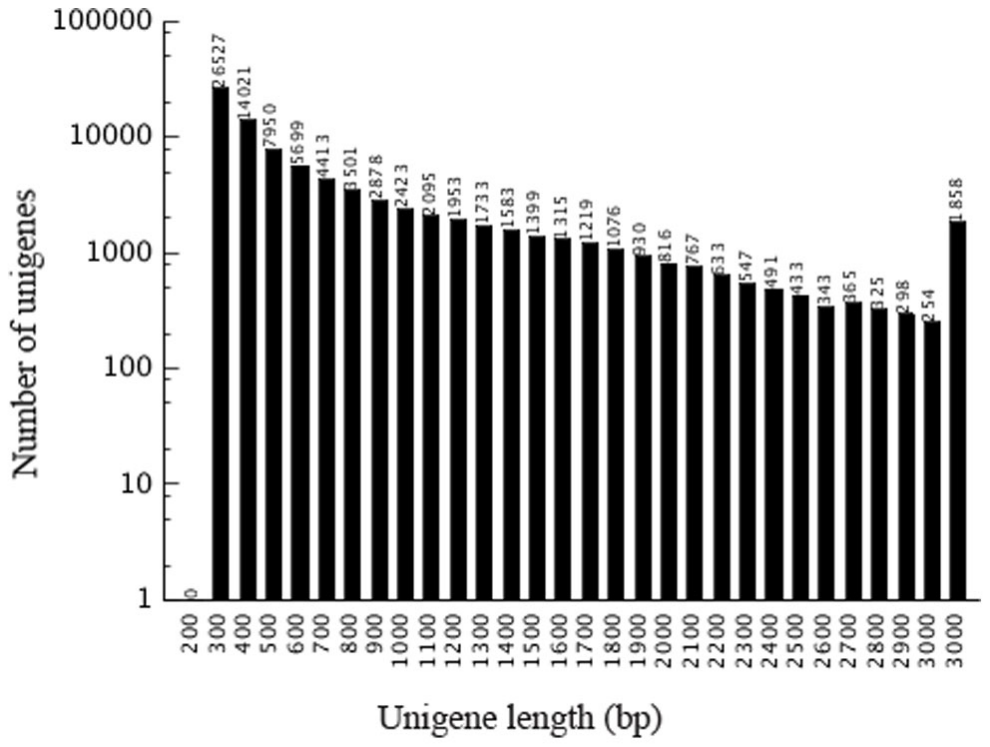
Length distribution of banana unigenes. The number of y-axis has been transferred into logarithmic scale.

A cDNA library for transcriptome analysis was prepared from the equally mixed RNA of roots of the susceptible cultivar ‘Brazilian’ and the resistant cultivar ‘Yueyoukang 1’. A total of 56.79 million 90-pair end raw reads (SRA accession number SRP026137) were generated through Illumina sequencing. To facilitate sequence assembly, repetitive, low-complexity and low-quality reads were filtered out. Reliable reads were assembled into 193,809 contigs with a mean length of 293 bp. After clustering the contigs together, we finally obtained 87,845 distinct sequences that cannot be extended on either end. Such sequences were defined as unigenes, including 33,532 clusters and 54,313 singletons, with a mean length of 755 bp. The size of each unigene was not less than 300 bp. And between 200 bp and 1000 bp, there were 67412 distinct sequences. The size distribution of these unigenes is shown in figure 1.

### Annotation

**Figure 2:**
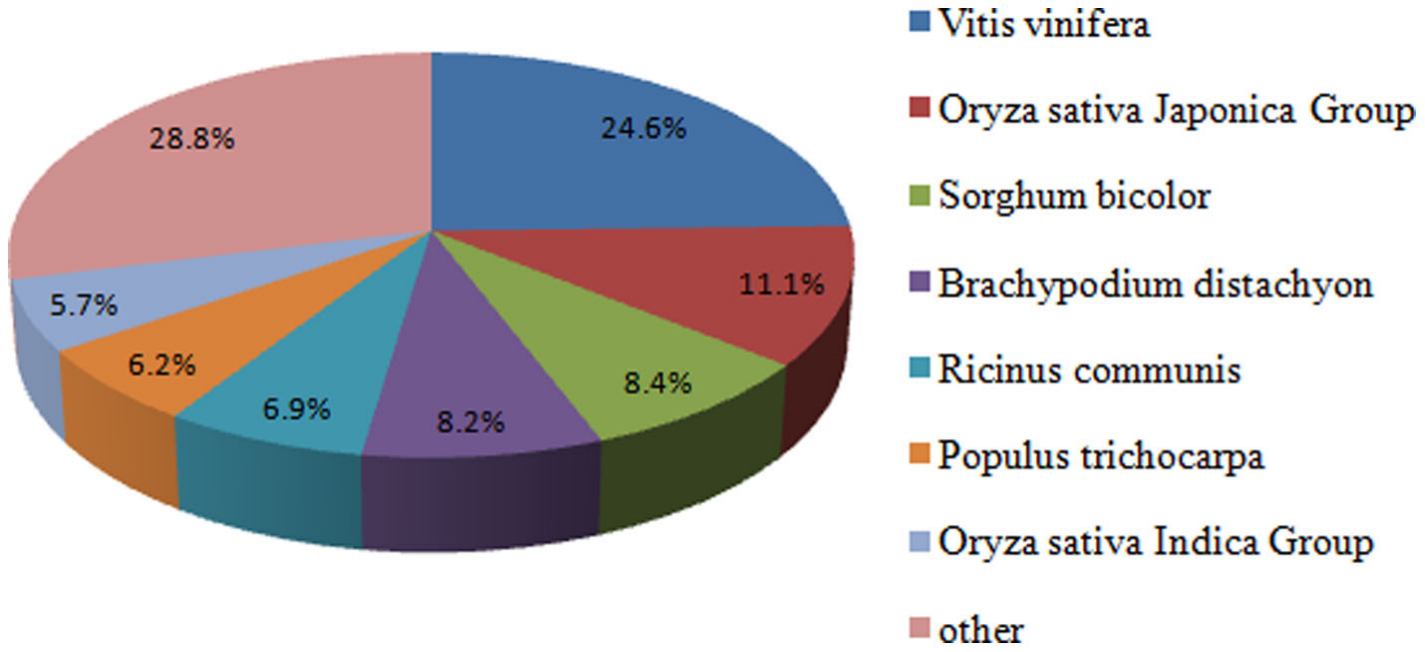
Species distribution of the top BLASTx hits. It shown as a percentage of the total homologous sequences with an E-value of at least 1.0E-5.

The annotation of 87,845 unigenes was based on the sequence similarity search in protein databases using BLAST algorithm. We first searched the reference sequences using BLASTX against the non-redundant (nr) NCBI protein database with a cut-off E-value of 10^−5^. A total of 59,340 sequences had good comparability with known gene sequences in existing species, which represented about 67.54% of the total unigenes. The species distribution of the best match result for each sequence is shown in figure 2. And 24.6% of the distinct sequences have top matches (first hit) with *Vitis vinifera*, followed by *Oryza sativa* Japonica Group (11.1%), *Sorghum bicolor* (8.4%), *Brachypodium distachyon* (8.2%), *Ricinus communis* (6.9%), *Populus trichocarpa* (6.2%), *Oryza sativa* Indica Group (5.7%). The proteins from NCBI nr database with the highest sequence similarity to the unigenes were used to assign functional annotations to the unigenes.

**Figure 3:**
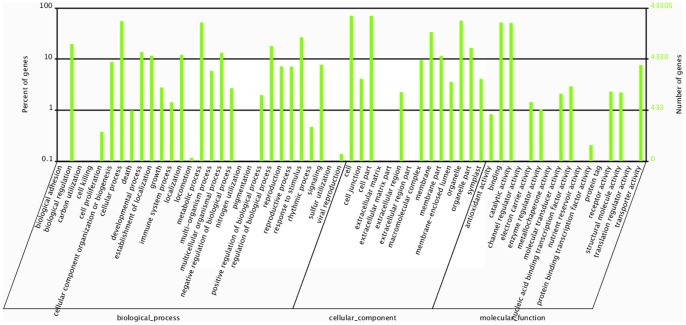
GO categories assigned to the banana unigenes.

Gene Ontology (GO) terms were assigned to banana unigenes based on their sequence matches to known proteins in the nr databases. A total of 43,305 unigenes, which were assigned with at least one GO term, can be categorized into 59 functional groups (Figure 3). Within the biological process, molecular function, and cellular component categories, the terms ‘cellular process and metabolic process’, ‘binding and catalytic activity’ and ‘cell, cell part, and organelle’ were dominant, respectively.

**Figure 4:**
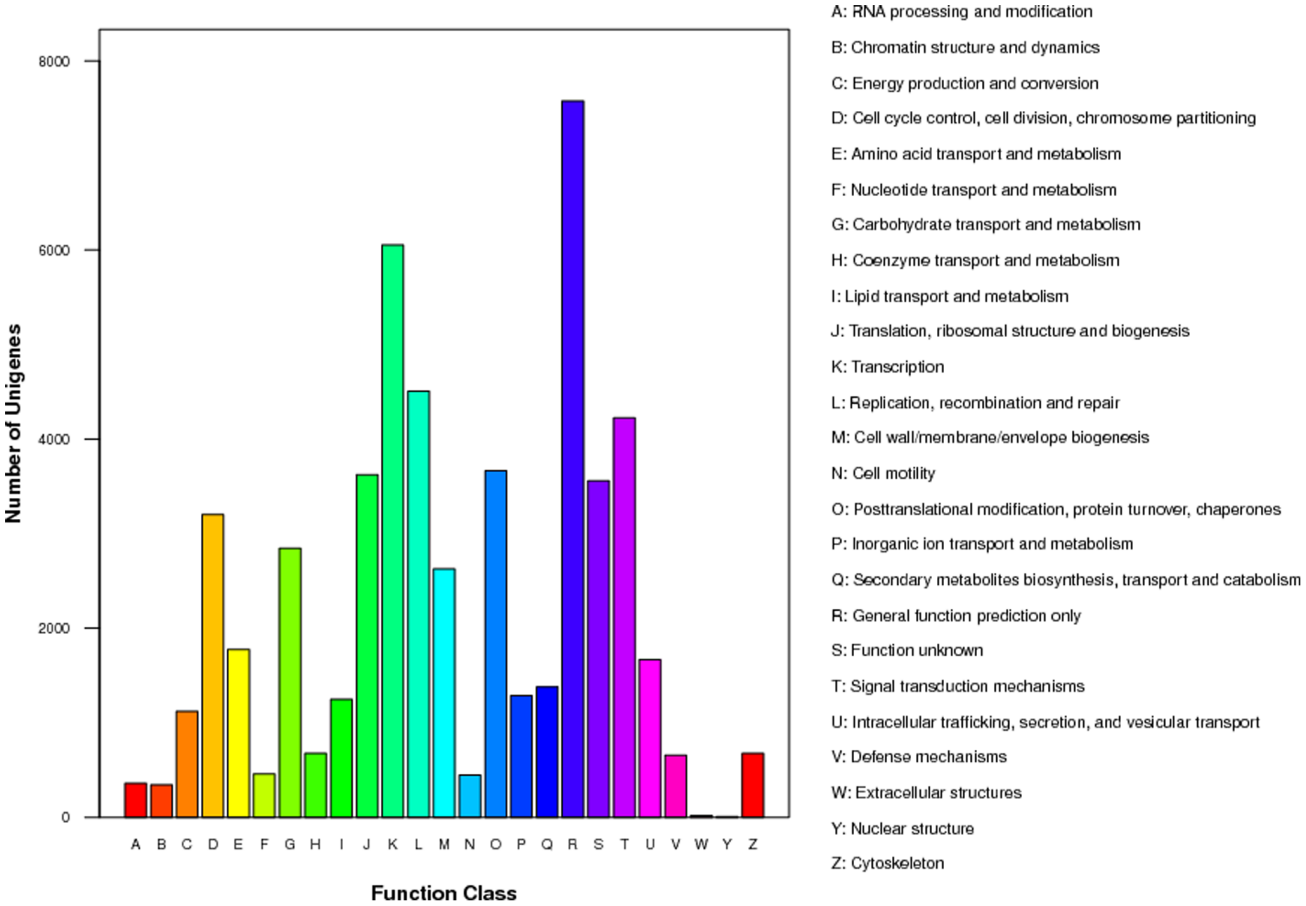
COG classification of banana unigenes after blastx search. The unigenes could be categorized into several function classes. Each function class was represented by different capital letters under the x-axis. The y-axis denotes the number of unigenes in a corresponding function class.

Using the COG databases to annotate the banana unigenes, we found 20,713 distinct sequences having a COG classification. Among the 25 COG categories, the largest group was ‘General function prediction’ (7,573), followed by ‘Transcription’ (6053), ‘Replication, recombination and repair’ (4506), and ‘Signal transduction mechanisms’ (4223). The smallest groups were ‘Nuclear structure’ (5) and ‘Extracellular structures’ (17) (Figure 4).

KEGG is a bioinformatics resource for linking genomes to life and the environment. The PATHWAY database records networks of molecular interactions in the cells, and variants of them specific to particular organisms. To identify the biological pathways that are active in the banana, we mapped the 59,340 unigene sequences to the reference canonical pathways in Kyoto Encyclopedia of Genes and Genomes. A total of 35,313 unigenes could be annotated in KEGG database and assigned to 127 KEGG pathways. The ‘metabolic pathway’ was the biggest term and contained 8747 unigenes occupying 24.77%, following by ‘biosynthesis of secondary metabolites’ (3073, 8.70%), ‘plant hormone signal transduction’ (2696, 7.63%), ‘endocytosis’ (2688, 7.61%), ‘Plant-pathogen interaction’ (2579, 7.30%) (Table 1).

**Table 1:** Top 20 enriched KEPP pathways.

Pathway	The number of DEGs	Pathway ID
Metabolic pathways	8747 (24.77%*)	ko01100
Biosynthesis of secondary metabolites	3073 (8.7%)	ko01110
Plant hormone signal transduction	2696 (7.63%)	ko04075
Endocytosis	2688 (7.61%)	ko04144
Plant-pathogen interaction	2579 (7.3%)	ko04626
Glycerophospholipid metabolism	2577 (7.3%)	ko00564
Ether lipid metabolism	2331 (6.6%)	ko00565
RNA transport	2273 (6.44%)	ko03013
mRNA surveillance pathway	1766 (5%)	ko03015
Spliceosome	1275 (3.61%)	ko03040
Protein processing in endoplasmic reticulum	928 (2.63%)	ko04141
Purine metabolism	850 (2.41%)	ko00230
Ribosome	768 (2.17%)	ko03010
Pyrimidine metabolism	748 (2.12%)	ko00240
Starch and sucrose metabolism	735 (2.08%)	ko00500
Ubiquitin mediated proteolysis	629 (1.78%)	ko04120
RNA degradation	596 (1.69%)	ko03018
Phenylpropanoid biosynthesis	562 (1.59%)	ko00940
Phosphatidylinositol signaling system	486 (1.38%)	ko04070
Ribosome biogenesis in eukaryotes	483 (1.37%)	ko03008

* The percentage of the total DEGs.

### Digital gene expression (DGE) library sequencing challenged with TR4

Total RNA was respectively extracted from roots of cv. ‘Brazilian’ and cv. ‘Yueyoukang 1’ after TR4 infection 0, 0.5, 1, 3, 5, and 10 DPI. Twelve DGE libraries (SRA accession number SRP026137) were sequenced and named BX, BX1, BX2, BX3, BX4, BX5 (from ‘Brazilian’), and YY, YY1, YY2, YY3, YY4, YY5 (from ‘Yueyoukang 1’), respectively. Each library generated about five million raw tags. After removing the low quality reads, the total number of clean tags in each library ranged between 4.3 and 4.8 million, occupying from 92.67 to 97.23%. All clean tags were mapped to our transcriptome reference database which was mentioned above. The percentage of these clean tags that could be mapped to the reference database ranged from 65.16 to 76.98%.

### Differentially expressed genes (DEGs) at Foc4 different infection stages

**Figure 5:**
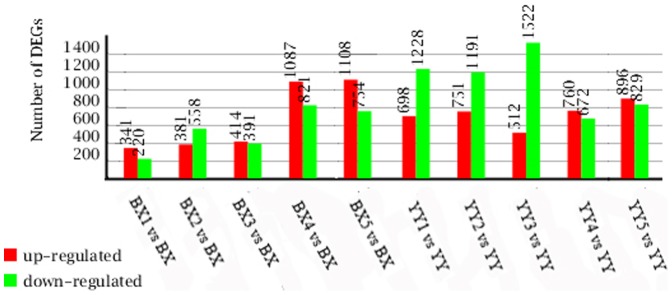
Numbers of DGE unigenes in each comparison. Up-(red) and down-regulated (green) unigenes were quantified. BX1 vs. BX, BX2 vs. BX, BX3 vs. BX, BX4 vs. BX and BX5 vs. BX respectively stand for 0.5, 1, 3, 5 and 10 DPI in ‘Brazilian’. YY1 vs. YY, YY2 vs. YY, YY3 vs. YY, YY4 vs. YY, vs. YY5 vs. YY respectively stand for 0.5, 1, 3, 5 and 10 DPI in ‘Yueyoukang 1’.

The differentially expressed genes (DEGs) of cv. ‘Brazilian’ and cv. ‘Yueyoukang 1’ at different TR4 infection stages were identified using an algorithm developed according to the method described by Audic et al [24]. We used “FDR≤0.001 and the absolute value of fold change (log2Ratio)≥1” as the threshold to judge the significance of gene expression difference [25]. The gene expression variations were analyzed between the comparisons of BX1 and BX, BX2 and BX, BX3 and BX, BX4 and BX, BX5 and BX, YY1 and YY, YY2 and YY, YY3 and YY, YY4 and YY, and YY5 and YY. The results revealed 341, 381, 414, 1087 and 1108 DEGs were up-regulated at 0.5, 1, 3, 5 and 10 DPI in ‘Brazilian’. And 220, 558, 391, 821 and 754 DEGs were down-regulated at 0.5, 1, 3, 5 and 10 DPI in ‘Brazilian’, respectively. While in ‘Yueyoukang 1’, 698, 751, 512, 760 and 896 DEGs showed up-regulated at 0.5, 1, 3, 5 and 10 DPI, respectively. The number of down-regulated were respectively 1228, 1191, 1522, 672 and 829 at 0.5, 1, 3, 5 and 10 DPI in ‘Yueyoukang 1’.The comparison results were combined and showed as figure 5.

### KEGG pathways analysis in both cv. ‘Brazilian’ and cv. ‘Yueyoukang 1’ after inoculation of TR4

We evaluated the TR4-affected biological pathways of susceptible and resistant banana by significantly enrichment analysis of DEGs. Pathways with Q value≤0.05 were regarded as significantly enrichment. 25 KEGG pathways showed significantly enrichment in resistant cv. ‘Yueyoukang 1’, of which 4 common pathways, namely ‘Metabolic pathways’ ‘Biosynthesis of secondary metabolites’ ‘Phenylpropanoid biosynthesis’ and ‘RNA transport’ were significantly enriched at all of the five infection stages. There were 13, 22, 8, 10 and 12 significantly enriched pathways for TR4 infection at 0.5, 1, 3, 5 and 10 DPI, respectively. The number of down-regulated DEGs was far more than up-regulated DEGs in almost all significantly enriched pathways at the first three time points. The remaining two time points contained most of significantly enriched pathways in which up-regulated DEGs were more than down-regulated DEGs. In susceptible cv. ‘Brazilian’, there were 13 significantly enriched pathways. Among these pathways, 3 common pathways including ‘Metabolic pathways’ ‘Phenylpropanoid biosynthesis’ and ‘Phenylalanine metabolism’ were found at all of the five infection stages. 0.5, 1, 3, 5 and 10 DPI respectively contained 5, 4, 9, 9 and 9 significantly enriched pathways. It was different from ‘Yueyoukang 1’ that the proportion of up-regulated DEGs in significantly enriched pathways was high in the first three time points of ‘Brazilian’. The time points of 0.5, 1 and 3 DPI respectively included 4, 2 and 3 significantly enriched pathways that had more up-regulated DEGs than down-regulated DEGs. And all the significantly enriched pathways of the following two time points contained more up-regulated DEGs than down-regulated DEGs. The summary of significantly enriched KEGG pathways influenced by Foc4 infection was shown in Table S2.

We also investigated the first ten top enriched pathways. Eight pathways, namely ‘Metabolic pathways’ ‘Biosynthesis of secondary metabolites’ ‘Endocytosis’ ‘RNA transport’ ‘Glycerophospholipid metabolism’ ‘Ether lipid metabolism’ ‘mRNA surveillance pathway’ and ‘Plant hormone signal transduction’ constituted the common top enriched pathways for the five infection stages of cv. ‘Yueyoukang 1’. The total number of DEGs in each common top enriched pathway ranged from 43 to 359. Other top enriched pathways were ‘Plant-pathogen interaction’ ‘Ribosome’ ‘Purine metabolism’ and ‘Phenylpropanoid biosynthesis’. ‘Plant-pathogen interaction’ with DEGs from 46 to 61 was involved in 1, 3, 5 and 10 DPI. ‘Ribosome’ with DEGs from 47 to 49 was involved in 0.5 and 3 DPI. ‘Purine metabolism’ with DEGs from 47 to 51 was involved in 0.5 and 1 DPI. ‘Phenylpropanoid biosynthesis’ with DEGs from 31 to 37 was involved in 5 and 10 DPI. ‘Brazilian’ had seven common top enriched pathways at all the five infection stages, including ‘Metabolic pathways’ ‘Biosynthesis of secondary metabolites’ ‘RNA transport’ ‘Endocytosis’ ‘Ether lipid metabolism’ ‘Glycerophospholipid metabolism’ and ‘mRNA surveillance pathway’. The total number of DEGs in each common top enriched pathway ranged from 23 to 342. Other top enriched pathways contained ‘Phenylpropanoid biosynthesis’ ‘Plant-pathogen interaction’ ‘Purine metabolism’ ‘Ribosome’ ‘Protein processing in endoplasmic reticulum’ and ‘Plant hormone signal transduction’. ‘Phenylpropanoid biosynthesis’ with DEGs from 15 to 45 was involved in 0.5, 3 and 10 DPI; ‘Plant-pathogen interaction’ with DEGs from 14 to 57 was involved in 0.5, 1, 5 and 10 DPI; ‘Purine metabolism’ with 13 DEGs was involved in 0.5 DPI; ‘Ribosome’ with DEGs from 25 to 34 was involved in 1, 3 and 5 DPI; ‘Protein processing in endoplasmic reticulum’ with 24 DEGs was involved in 1 DPI; ‘Plant hormone signal transduction’ with DEGs from 25 to 54 was involved in 3, 5 and 10 DPI (Table S3 and Table S4).

### qRT-PCR validation

To confirm the results of the Illumina Solexa sequencing, eight unigenes were randomly selected from those listed in Table S6 for quantitative RT-PCR assays. These unigenes were related to CEBiP, CERK1, Rboh, PBS1, WRKY22, PR-1, MYC2 or CCoAM. All of these unigenes showed significantly differential expression at some time points and no differential expression at the other time points. We found that qRT-PCR validation of one unigene (Unigene15900_BXA-1 annotated as CERK1) which showed significantly down-regulated at 3, 5 and 10 DPI in ‘Yueyoukang 1’ and significantly up-regulated at 5 DPI in ‘Brazilian’ was not consistent with DGE analyses. For the other seven unigenes, qRT-PCR revealed the same expression tendency as the DGE analyses (Table S5). This further suggested that the Illumina Solexa sequencing in this study was of high reliability.

## Discussion

Bananas and plantains (*Musa spp.*) are one of the most important fruit crops in the world. Recently, banana draft genome sequencing was completed but the genome sequences are still not released [26]. In this report, we utilized Illumina Solexa sequencing technology, a high- throughput DNA sequencing approach, to performed transcriptome sequencing of banana. The banana cDNA library used in this research was constructed from the equally mixed mRNA of roots of the healthy susceptible cultivar ‘Brazilian’ and highly resistant cultivar ‘Yueyoukang 1’. After Illumina sequencing and sequence assembly, we finally obtained 87,845 distinct sequences, of which 59,340 sequences had good comparability with known gene sequences in the non-redundant (nr) NCBI protein database with a cut-off E-value of 10^−5^. Functional annotation analysis revealed that 43,305 and 20,713 unigenes could be annotated in GO and COG databases, respectively. KEGG annotation analysis showed a total of 35,313 unigenes could be annotated in KEGG database and assigned to 127 KEGG pathways. The top 20 enriched KEGG pathways were shown as Table 1. The first five pathways contained metabolic pathway’, ‘biosynthesis of secondary metabolites’, ‘plnat hormone signal transduction’, ‘endocytosis’ and ‘Plant-pathogen interaction’. In order to identify the quality of our sequencing data, we randomly chose 8 unigens and designed 8 pairs of primers for RT-PCR amplification. The result that 7 out of 8 primer pairs resulted in an expected size band suggests that our transcriptome sequences have a high reliability (data not shown).

To better understand the gene expression differences between susceptible and resistant banana cultivar challenged with Foc TR4, we constructed 6 digital gene expression (DGE) libraries for each cultivar during different TR4 infection stages (0, 0.5, 1, 3, 5 and 10 DPI). DGE technology provides a quantitative measure of transcript abundance and has a higher precision than microarrays [27]. It has been used to detect differences in gene expression between two different samples. Here, we generated about five million raw tags from each library. Comparing with the 0 DPI, we identified differentially expressed genes (DEGs) of 0.5, 1, 3, 5 and 10 DPI for each cultivar. The total number of DEGs in resistant ‘Yueyoukang 1’ was far more than that in susceptible ‘Brazilian’ at the first three infection time points (Figure 5). KEGG pathway analysis revealed that DEGs mainly distributed in 11 pathways including ‘Metabolic pathways’, ‘Biosynthesis of secondary metabolites’, ‘Endocytosis’, ‘RNA transport’, ‘Glycerophospholipid metabolism’, ‘Ether lipid metabolism’, ‘mRNA surveillance pathway’, ‘Plant hormone signal transduction’, ‘Plant-pathogen interaction’, ‘Ribosome’ and ‘Phenylpropanoid biosynthesis’. These pathways were associated with metabolism, transport, transcription, lipid metabolism, signal transduction and defense, which participate in regulation of responses to pathogen attack [10], [28]. The number of DEGs in ‘Plant-pathogen interaction’ pathway was respectively 47, 61, 57, 46 and 57 in resistant ‘Yueyoukang 1’ banana at 0.5, 1, 3, 5 and 10 DPI, and it was respectively 14, 23, 24, 57 and 55 in susceptible ‘Brazilian’ banana at these same time points. These results suggested that ‘Yueyoukang 1’ had much faster response to TR4 infection than ‘Brazilian’. Genes involved in the plant-pathogen interaction pathway form a specific system with multiple layers against pathogen infection [29]. And in the system, pattern-recognition receptors (PRRs) by cell-surface can recognize pathogen-associated molecular patterns (PAMPs) to activate the PAMP-triggered immunity (PTI) [30]. The elevation of cytosolic Ca^2+^ concentration regulates reactive oxygen species production and is involved in programmed cell death [31], [32]. Although pathogens can acquire the ability to suppress PTI by directly injecting effector proteins into the plant cell through secretion systems, NB-LRR immune receptors in plant have evolved the ability to recognize effector and initiate effector-triggered immunity (ETI) [33]. The signals mediated by PRRs and NB-LRR immune receptors are transduced through mitogen-activated protein kinase (MAPK) cascades and transcription factors which further activate immune responses. These plant responses to pathogen attack mainly include hypersensitive reaction (HR)/programmed cell death, expression of pathogenesis-related (PR) proteins, cell wall lignification, and so on [34]. In addition, the number of DEGs in ‘plant hormone signal transduction’ pathway was far more in resistant ‘Yueyoukang 1’ than that in susceptible ‘Brazilian’ at the first three time points (Supplemental tables S3 and S4). Previous work also reported that plant hormones play a role in plant defense response to biotic stress [35]. In our results, the expression patterns of these genes were different between highly resistant ‘Yueyoukang 1’ and susceptible ‘Brazilian’ challenged with TR4, some of which were not consistent with observation in ‘Nongke No 1’ banana [15]. Detailed description is shown in Supplemental Table S6 and as follows.

### Pattern Recognition Receptors (PRRs)

Pattern recognition receptors (PRRs) detect the conserved pathogen-associated molecular patterns (PAMPs) and trigger plant immunity to protect host from pathogen infection. Therefore PRRs play a fundamental role in PAMP-triggered immunity(PTI) [30]. In our expression profile results, most of PRRs gens showed different expression patterns between resistant ‘Yueyoukang 1’ and susceptible ‘Brazilian’ in response to TR4 infection. Chitin elicitor-binding protein (CEBiP) can recognize chitin oligosaccharides on the fungal cell surface and induce defense response in plant cells, and knockdown of the CEBiP gene resulted in marked suppression of chitin elicitor-induced defense response [36]. Our study revealed that CEBiP was significantly induced in ‘Yueyoukang 1’ at the first four time points, while it was only induced in ‘Brazilian’ at 5 and 10 DPI, which is in agreement with the report that the expression of CEBiP were up-regulated in middle resistant ‘Nongke No 1’ compared to ‘Brazilian’ 2 and 4 days after TR4 infection [15]. The results suggested that TR4 infection trigger an early immune response in resistant cv. ‘Yueyoukang 1’ compared to the susceptible cv. ‘Brazilian’ and CEBiP confers resistance to TR4 infection. Chitin elicitor receptor kinase (CERK1), a partner of CEBiP, transduces signal to trigger PTI in rice [37]. The genes related to CERK1 exhibited down-regulated in ‘Yueyoukang 1’ but up-regulated in ‘Brazilian’ at the first three time points. The result was out of our expectation. Maybe CERK1 did not play a role in incompatible interaction between ‘Yueyoukang 1’ banana and TR4.

Brassinsteroid insensitive 1-associated receptor kinase 1 (BAK1) is another significant pattern recognition receptor in plant. It was required for PTI triggered by bacterial flagellin and elongation factor Tu. Flagellin-sensitive 2 (FLS2), a leucine-rich repeat receptor kinases, was characterized as PRR for the bacterial flagellin. FLS2 and BAK1 form a complex to initiate innate immunity in plant [4], [38], [39]. Although no literature reported that BAK1 was involved in PTI triggered by fungus, our results revealed that the number of DEGs related to BAK1 was the most extensive in all kinds of the PRRs. And the induced BAK1 gene in the resistant cv. ‘Yueyoukang 1’ was up to a 477.7-fold increase, which was much higher than that in ‘Brazilian’. As reported in the ‘Nongke No 1’ [15], the results indicated that BAK1 played a role in the resistance response against TR4 infection. The results is consistent with that BAK1 also control plant programmed cell death and immunity to necrotrophic fungi, and bark1 mutants of *Arabidopsis* showed extreme susceptibility to necrotrophic fungi [40]. Genes related to FLS2 showed down-regulated in resistant ‘Yueyoukang 1’ but up-regulated in susceptible ‘Brazilian’ at the first three time points and this expression pattern was similar to CERK1, which suggested FLS2 did not play a role in incompatible interaction between banana and TR4.

### DEGs related to CNGC and Rboh

Ca^2+^ is utilized as an important secondary messenger in signaling cascades by plant cell and plays an essential role for production of hypersensitive reaction in plant response to biotic stress [32]. AtCNGC2, 4, 11, and 12 have been identified to be involved in plant immunity [41]. In our expression profile results, however, we found that three kinds of CNGC including CNGC1 decreasing at 1 DPI in ‘Brazilian’, CNGC4 increasing at 5 and 10 DPI in ‘Brazilian’, and CNGC5 decreasing at 10 DPI in ‘Yueyoukang 1’ showed significantly differential expression. This suggested that Ca^2+^ influx changed in banana response to Foc4 infection. CNGC4 was induced in response to pathogen attack and might participate in signaling pathways leading to HR [42]. HR might be targeted by non-biotrophic fungal pathogens during successful colonization [43]. Another component which plays a central role in the development of HR in plant attacked by pathogen is reactive oxygen species (ROS) [44]. Respiratory burst oxidase homolog (Rboh) gene participating in production of ROS was found to be up-regulated in ‘Brazilian’ at 5 and 10 DPI. While in ‘Yueyoukang 1’, Rboh expression level had no any change at 5 and 10 DPI and just was suppressed by TR4 at 0.5, 1 and 3 DPI. It was been reported that germinating TR4 spores were observed to colonize ‘Brazilian’ banana roots at 5 DPI [7]. Hence we guessed CNGC4 and Rboh were induced in ‘Brazilian’ by TR4 to produce HR which may benefit TR4 further infection. However, this hypothesis needs to be further examined. It was worth noting that our results were not consistent with previous report by Li, in which only the expression of CNGC 1, CNGC 5 and CNGC 6 changed in response to TR4 infection and Rboh were up-regulated in the middle resistant ‘Nongke No 1’ banana in response to TR4 infection compared with ‘Brazilian’ [15]. Maybe the different results were caused by the difference between two resistant cultivars and the different time points of TR4 infection.

### NB-LRR disease resistance protein

The majority of R proteins in plant containing cytoplasmic nucleotide-binding site (NB) and leucine-rich repeat (LRR) domains confer resistance to diverse pathogens such as bacteria, fungi, viruses, nematodes and insects [45]. In our research, RPM1 and PBS1 both belonging to NB-LRR genes were strongly induced in ‘Yueyoukang 1’ after TR4 inoculation in contrast to ‘Brazilian’, which was in agreement with previous observation in the resistant ‘Nongke No 1’ banana [15]. And mRNA level of RPM1 in ‘Yueyoukang 1’ started to increase at 0.5 DPI, which was earlier than that in ‘Brazilian’. It was reported that RPM1 confer resistance to disease caused by *Pseudomonas syringae* strains expressing *avrRpm1* or *avrB* effector [46]. Previous work also showed that PBS1 can be cleaved by *Pseudomonas syringae* effector *AvrPphB* and the cleavage activates RPS5 to initiate ETI immunity in *Arabidopsis* [47]. Hence RPM1 and PBS1 may be required to mediate incompatible interactions in banana. RPM1-interacting protein 4 (RIN4) showed down-regulated in both cultivars after challenged with TR4. The expression of RIN4 was repressed as early as 12 hour after TR4 inoculation, which also was earlier than that in ‘Brazilian’. Although RIN4 is required for RPM1 accumulation to mediate disease resistance, on the other hand RIN4 disappearance activates RPS2-mediated disease resistance pathway [48]. The effector secreted by Foc is still unknown. To better understand the mechanism about how RIN4 regulates disease resistance mediated by RPS2 and RPM1 in banana challenged with TR4, more work remains to be done.

### Transcription Factors

Plant transcription factors participated in regulation of the expression of defense-related genes against different types of microbial pathogen. In our research, three transcription factors including WRKY transcription factor 22, 33 and DREB protein showed different expression pattern between ‘Yueyoukang 1’ and ‘Brazilian’. In ‘Yueyoukang 1’, two WRKY22 genes were up-regulated and one was down-regulated at both first two time points; three, one and three WRKY22 genes showed up-regulated at 3, 5 and 10 DPI, respectiv*ely*. However, no any WRKY22 gene showed differently expressed in ‘Brazilian’ until 5 DPI. The number of up-regulated WRKY33 genes was more and the down-regulated was less in ‘Yueyoukang 1’ than ‘Brazilian’ at almost all the five time points. And up-regulated WRKY33 exhibited higher increase level in ‘Yueyoukang 1’ than ‘Brazilian’ at the first two time points. DREB protein was mainly up-regulated at 3, 5 and 10 day after inoculation and its increase level was very higher in ‘Yueyoukang 1’ than ‘Brazilian’. They have been documented to play an important role in resistance response to pathogen attack [49]–[51]. Especially WRKY33 is required for resistance to necrotrophic fungal pathogens [50]. These results firstly suggested that these transcription factors may be involved in plant process against TR4 infection in banana. In addition, TGA transcription factor involved in SA-mediated defense showed that higher expression level in the resistant cv. ‘Yueyoukang 1’ in contrast to the susceptible cv. ‘Brazilian’. TGA is known to function in the regulation of pathogenesis related genes and defense response [52]. And it was also reported to participate in Pro-mediated disease resistance in tomato [53].

### Defense Response Genes

One of the features of plant defense response is production of pathogenesis-related (PR) proteins that have been widely studied in plant pathogen resistance. In our research, the constitutive expression of glucanases and chitinases was significantly higher in cv. ‘Yueyoukang 1’ compared with the susceptible cv. ‘Brazilian’. When inoculated with the FOC4, gene expression of pathogenesis-related (PR)-1, glucanases and chitinases were affected in both cultivars. PR-1 was mainly up-regulated in the resistant ‘Yueyoukang 1’ and down-regulated or changeless in the susceptible ‘Brazilian’ at the first three time points. However, PR-1 mainly showed down-regulated in ‘Yueyoukang 1’ but up-regulated in ‘Brazilian’ at the last two time points, suggesting that PR-1 may be involved in the incompatible interaction at the early infection stages. The expression of glucanase changed dramatically over time in both cultivars. Glucanase genes were highly expressed in the resistant cv. ‘Yueyoukang 1’ at all time points compared with those in the susceptible cv. ‘Brazilian’. Chitinase gene was up-regulated starting at 1 DPI and came to the highest level at 5 DPI in ‘Yueyoukang 1’, while it showed up-regulated only at 1 and 10 DPI in the susceptible cv. ‘Brazilian’. The induction and greater accumulation of mRNA of PR-1, glucanases and chitinases in ‘Yueyoukang 1’ after TR4 infection suggested that these genes were associated with plant defense in banana roots. This was consistent with the previous study which reported that PR-1, glucanases and chitinases might serve to resist Foc in banana [54].

### DEGs Involved in Salicylic Acid, Jasmonic Acid and Ethylene Pathway

It has been reported that salicylic acid, jasmonic acid and ethylene play important roles in signaling networks and regulation developmental processes in plant response to biotic stress [35]. In our results, benzoic acid 2-hydoxylase and isochorismate synthase required for production of SA did not significantly change in both cultivars. However, the SA signaling related genes such as TGA transcription factor, WRKY transcription factor and PR-1 protein mentioned above were higher induced by TR4 in ‘Yueyoukang 1’ compared with ‘Brazilian’. Plant hormone signaling pathways and other signaling proteins constitute complex networks of interactions regulated by signaling molecules. Pathogen attack triggers complex signaling cascades in these networks, resulting in the expression of defense-related genes such as those encoding PR proteins. mRNA levels of lipoxygenases (LOX) and allene oxide synthase (AOS) needed to JA production showed up-regulated in the susceptible cv. ‘Brazilian’ at all time points, while they were down-regulated in the resistant cv. ‘Yueyoukang 1’ at the first four time points. The result is not consistent with previous report that the expression levels of LOX and AOS were much higher in middle resistant ‘Nongke No 1’ than ‘Brazilian’ [15]. COI1, JAZ and MYC2, the downstream genes in JA signaling pathway, had the similar expression pattern with LOX and AOS, which were up-regulated in ‘Brazilian’ and down-regulated in ‘Yueyoukang 1’. Senescence is associated with increased JA levels in *Arabidopsis*, and treatment of *Arabidopsis* with JA induces COI1-dependent senescence [55]. Meanwhile increased senescence leads to susceptibility to *Fusarium oxysporum* in *Arabidopsis* [56]. It was also reported that *Fusarium oxysporum* hijacks COI-mediated JA-signaling pathway to cause wilt-disease symptoms that lead to plant death in *Arabidopsis* [57]. Therefore, TR4 perhaps captures the non-defensive aspects of the JA-signaling pathway, alter senescence, and increase susceptibility in ‘Brazilian’. This may be why ‘Brazilian’ is highly susceptible to TR4 but ‘Yueyoukang 1’ is resistant to TR4. Ethylene biosynthetic and signaling related genes exhibited no significant differences in roots of both cultivars response to TR4 infection, which indicated that ethylene did not play a role in the resistance response.

### Cell Wall Lignification

Plant cell wall forms the first plant barriers to pathogen infection. On the one hand, plant cell wall acts as physical barrier to protect the whole cell. On the other hand, plants may perceive pathogen attack by sensing cell wall integrity [58]. 4-coumarate: CoA ligase (4CL), glutathione S-transferase (GST), cellulose synthase, Caffeoyl-CoA O-methyltransferase (CCoAM), and cinnamylalcohol dehydrogenase (CAD) are involved in the synthesis of lignin polymers and reinforcement of the wall. Investigation found that 4CL, GST, CCoAM and CAD displayed an increase in levels of mRNA in TR4-treated roots of resistant cv. ‘Yueyoukang 1’. While in susceptible cv. ‘Brazilian’, only the expression of cellulose synthase and CCoAM were induced at all time points. mRNA levels for GST were decreased at 0.5 DPI but were later increased at 1, 5 and 10 DPI. CAD gene only showed up-regulated at 3 DPI. mRNA of these genes were increased following TR4 treatment, suggesting that lignification is one of the mechanisms during the banana defense response to resist TR4 infection in both resistant and susceptible cultivar. Further analysis showed that the transcription levels of GST, CCoAM and CAD in the highly resistant cultivar were higher than those in the susceptible cultivar, which is in agreement with previous observation in watermelon challenged with *Fusarium oxysporum* f. sp. *Niveum* [59]. These results indicated that full lignification is likely to contribute to disease resistance. However, the results are also not consistent with the previous report that genes associated with lignification did not change or were repressed in wilt-resistant banana cv. ‘Nongke No 1’ [15]. The possible reason might be that the pathogen-resistance mechanism is different between the two cultivars.

## Conclusion

In this study, we performed transcriptome and expression profiles sequencing of the highly resistant ‘Yueyoukang 1’ and the susceptible ‘Brazilian’ banana using Illumina technology, and compared expression profile differences between the two cultivars infected by Foc TR4. The results showed that genes related to CEBiP, BAK1, NB-LRR proteins, PR proteins, transcription factor and cell wall lignification were induced in ‘Yueyoukang 1’ banana, suggesting that these genes play important roles in the incompatible interactions between banana and Foc TR4. Meanwhile those genes controlling hypersensitive reaction and senescence, such as CNGC4, Rboh and genes in jasmonic acid pathway, were suppressed by TR4 in ‘Yueyoukang 1’ but were induced by TR4 in ‘Brazilian’, suggesting that TR4 may induce this response as part of its infection strategy. These genes initially identified by the method of comparative transcriptome analyses still require further research to determine their functions and dissect the complex interacting signaling networks. Some of defense-related genes show different expression patterns between highly resistant ‘Yueyoukang 1’ and middle resistant ‘Nongke No 1’ challenged with Foc TR4, suggesting that the two cultivars had different pathogen-resistance mechanism. In conclusion, our study expands the transcript information of banana, explains the resistance in the highly resistant cv. ‘Yueyoukang 1’ and gains more insights into banana-necrotrophic pathogen interactions. However, many genes involved in the incompatible interactions between banana and Foc4 still remain unknown. In the future, more work will be required to decipher the true nature of banana wilt resistance and successfully control banana wilt disease.

## Supporting Information

Table S1 **Primers used in qRT-PCR for validation of differentially expressed genes.**
(XLS)

Table S2 **Significantly enriched KEGG pathways in ‘Yueyoukang 1’ and ‘Brazilian’.**
(XLS)

Table S3 **The first ten top enriched pathways in ‘Yueyoukang 1’.**
(XLS)

Table S4 **The first ten top enriched pathways in ‘Brazilian’.**
(XLS)

Table S5 **Verification of DGE analysis results by qRT-PCR.**
(XLS)

Table S6 **Differentially expressed genes related to plant resistance.**
(XLS)
